# Ultralow Threshold Room Temperature Polariton Condensation in Colloidal CdSe/CdS Core/Shell Nanoplatelets

**DOI:** 10.1002/advs.202200395

**Published:** 2022-04-24

**Authors:** Hongyu Yang, Lei Zhang, Wenbin Xiang, Changgui Lu, Yiping Cui, Jiayu Zhang

**Affiliations:** ^1^ Advanced Photonics Center Southeast University Nanjing Jiangsu 210096 P. R. China; ^2^ School of Sciences Nantong University Nantong 226019 P. R. China

**Keywords:** coherent light source, colloidal nanoplatelets, polariton BEC, strong exciton‐photon coupling

## Abstract

Room‐temperature exciton‐polariton Bose‐Einstein condensation (BEC), a phase transition to single quantum state with strong nonlinearity, provides a new strategy for coherent light sources and ultralow threshold optic switches. In this work, colloidal CdSe/CdS 2D nanoplatelets are embedded into a microcavity, and exciton‐polariton BEC is realized with an ultralow threshold of 0.5 µJ cm^–2^ at room temperature. The superlinear power‐dependent emission, macroscopic occupation of the ground state, strong blueshift and broadening of the emission peak, and long‐range coherence strongly confirm the realization of the polariton laser. This work suggests considerable prospects for colloidal nanoplatelets in low‐cost, high‐performance polariton devices, and coherent light sources.

## Graphical abstract

Room temperature polariton Bose–Einstein condensation is realized for the first time in colloidal CdSe/CdS nanoplatelet system with an ultralow threshold of 0.5 μJ cm^−2^. The build‐up of spontaneous long‐range spatial and temporal coherence indicates a new strategy for coherent light sources. In addition, this work also provides a new physical platform for the ultralow threshold quantum devices.

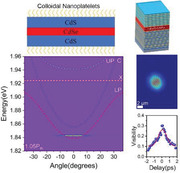

## 1 Introduction

Exciton‐polaritons are a kind of Bose quasiparticles resulted from the strong coupling between cavity photons and excitons.^[^ ^1^ ^]^ The half‐light and half‐matter nature of exciton‐polaritons gives rise to their extremely light effective mass, typically only 10^–4^–10^–5^ times that of an electron, which is considered to play an essential role in realizing Bose–Einstein condensation (BEC) at high temperature. Exciton‐polariton BEC or lasing, which results from the bosonic many‐body effect, leads to ultralow threshold macroscopic ground state coherent occupation.^[^ ^1^, ^2^ ^]^ In addition, since polariton lasing is attributed to bosonic final‐state stimulation, population inversion is not a prerequisite,^[^ ^3^ ^]^ which is crucially different from the conventional photonic laser. Therefore, polariton lasers have a significantly lower threshold than vertical cavity surface emitting lasers (VCSELs) despite having the same structure.^[^ ^4^ ^]^

Polariton laser was first demonstrated in the solid‐state systems of CdTe^[^ ^2^, ^5^ ^]^ and GaAs^[^ ^6^ ^]^ inorganic quantum wells. However, excitons are easily dissociated into electron–hole plasma due to their small Wannier exciton binding energy. Therefore, BEC is limited at cryogenic temperature. In contrast, polariton condensation in inorganic systems can be realized at room temperature with ZnO^[^ ^7^ ^]^ and GaN^[^ ^8^ ^]^ benefiting from their large exciton binding energy. Nonetheless, the construction of high‐performance microcavities with these inorganic gain materials is very challenging since they usually require precise epitaxial control. In contrast, organic materials exhibit Frenkel exciton with large binding energy and are easy to be fabricated, which provide substitutes for realizing room temperature polariton emission and condensation, such as, boron‐dipyrromethene fluorescent dye(BODIPY‐G1),^[^ ^9^ ^]^ Methyl‐substituted ladder‐type poly(p‐phenylene)(MeLPPP)^[^ ^10^ ^]^ and 2,7‐Bis[9,9‐di(4‐methylphenyl)‐fluoren‐2‐yl]‐9,9‐di(4‐methylphenyl)fluorene(TADF).^[^ ^11^ ^]^ However, the weak Coulomb interaction and small oscillator strength of Frenkel excitons in organic materials limit the polariton–polariton interaction, which plays the key role in polariton BEC, leading to inefficient condensation to the ground state. Therefore, the weak nonlinearity and high threshold (10^1^–10^2^ µJ cm^–2^) of the organic system have obstructed their development.

2D CdSe‐based colloidal semiconductor inorganic nanoplatelets have been extensively studied recently owing to their high photoluminescence quantum yield, parabolic shape exciton band similar to those of traditional quantum wells, long gain lifetime(>400 ps), giant gain coefficients (>6000 cm^–1^), and large absorption cross section (~10^–13^ cm^–2^).^[^ ^12^ ^]^ Due to the strong quantum confinement effect in the out‐of‐plane direction, the exciton binding energy can be as high as 100–200 meV,^[^ ^13^ ^]^ therefore, the excitons in nanoplatelets are highly stable at room temperature. In addition, the ground state exciton has a giant oscillator strength (15–23 Debye) due to the exciton center of mass coherent motion.^[^ ^14^ ^]^ Therefore, nanoplatelets are beneficial for studying strong light‐matter coupling in microcavities. Lucas et al.^[^ ^15^ ^]^ observed the anti‐crossing behavior of polariton using CdSe nanoplatelets and a tunable planar microcavity with which the coupling constant was estimated to be 33 meV. Shlesinger et al.^[^ ^16^ ^]^ obtained a Rabi‐splitting energy of 47 meV by analyzing the exciton‐plasma polariton dispersion with the sample composed of a gold film and CdSe/CdS core/shell nanoplatelets. In addition, the energy level structure of the nanoplatelets is only related to the thickness, and the fluorescence peak position and the single nanoplatelet spectrum at room temperature are nearly the same as those observed in the ensemble.^[^ ^17^ ^]^ Therefore, the inhomogeneous broadening of nanoplatelets can be eliminated differently from colloidal quantum dots. These excellent properties of nanoplatelets imply that this material is a powerful physical platform for studying polariton BEC at room temperature, and has great application potential in devices such as low‐threshold lasers, optical switches, and nonlinear logic gates.

In this work, we have successfully prepared colloidal CdSe/CdS core/shell nanoplatelets and embedded them into a Fabry‐Perot microcavity with high‐reflectivity distributed Bragg reflectors. The strong coupling between heavy‐hole/electron excitons and microcavity photons was confirmed by angle‐resolved reflectivity and photoluminescence spectroscopy. The microcavity was then pumped with a femtosecond laser to study the nonlinear properties. The polariton BEC occurs when the pump power exceeds 0.5 µJ cm^–2^, manifested as a robust superlinear emission intensity, the blueshift of the emitted photon energy, and the broadening of the spectra with the increase of pump power. The unambiguous polariton lasing was further confirmed by the long‐range spatial coherence and long temporal coherence (1.26 ps). For the first time, this study demonstrated the exciton–polariton BEC at room temperature in colloidal nanoplatelets system, which is of substantial research significance and great value to the further development of the nanoplatelet‐based optical devices.

## 2 Results and Discussion

### 2.1 Nanoplatelet Characteristics

**Figure 1:**
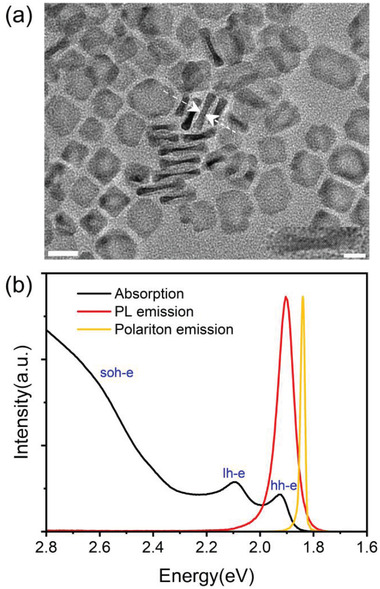
a) TEM image of CdSe/CdS nanoplatelets with both edge‐up(pointed) and face‐down orientations (large lateral area). Scale bar: 20 nm. The inset shows the HRTEM image. Scale bar: 5 nm. b) Room temperature absorption and PL spectra. Black line, the absorption spectrum of the CdSe/CdS nanoplatelets in hexane solution, showing three exciton transitions: hh‐e, lh‐e, and soh‐e at 1.924, 2.096, and 2.606 eV, respectively. Red line, the PL spectrum of the CdSe/CdS nanoplatelets in hexane solution, showing emission at 1.904 eV with FWHM of 64 meV. Yellow line, the ground‐state emission of the nanoplatelets embedded into the microcavity, showing an emission located at 1.840 eV with FWHM of 27 meV.

The colloidal nanoplatelets consisted of CdSe with four monolayers (MLs) thick as core and 6MLs CdS shell were prepared through a hot‐injection method (see Methods). The CdS shell provides efficient passivation on the dangling bonds of the CdSe core, which could thus increase the photoluminescence quantum yield (≈65%) and photostability.^[^ ^17^, ^18^ ^]^ The thickness of the nanoplatelets is estimated to be 4.7 nm by analyzing the transmission electron microscope (TEM) image with edge‐up orientation in **Figure** **1**a. The crystal lattice is visible in the high‐resolution TEM (HRTEM), demonstrating that these nanoplatelets are highly crystallizable. The nanoplatelet solution UV–vis absorption and PL spectra at room temperature are shown in Figure 1b. The absorption spectrum shows three well‐resolved transitions located at 1.924, 2.096, and 2.606 eV, which is similar to the traditional quantum well that corresponds to the heavy‐hole/electron exciton (hh‐e), the light‐hole/electron exciton (lh‐e) and the spin–orbit‐hole/electron exciton(soh‐e) transitions.^[12a]^ The PL peak is located at 1.904 eV with full‐width at half maximum (FWHM) of 64 meV. This narrow linewidth material is very suitable for polariton construction. The absorption, PL spectra, and refractive index of the nanoplatelet film desposited on a quartz slide were also measured, as shown in Figure S1 (Supporting Information), which are consistent with those of the solution besides a slight redshift, indicating that the excitonic feature is preserved in the film.

### 2.2 Linear Polariton Property

The scheme of the microcavity is shown in Figure S2 (Supporting Information), which consists of a nanoplatelet film with a thickness of ≈1 µm (made by drop‐casting from dense solution) embedded in the bottom distributed Bragg reflector (DBR) made of 12 alternating pairs of titanium dioxide/silicon dioxide (TiO_2_/SiO_2_) and top DBR made of eight alternating pairs zinc sulfide/calcium fluoride (ZnS/CaF_2_; see Experimental Section). These DBRs have high reflectivity of more than 99%, ensuring strong out‐of‐plane(k_⊥_) confinement in the photonics bandgap. Polariton is then formed resulting from the strong coupling of microcavity photon and nanoplatelets hh‐e excitons. Figure 1b shows the polariton ground state emission, from which we derive the FWHM of 16 meV, corresponding to the microcavity quality‐factor (Q‐factor) of 115.

**Figure 2:**
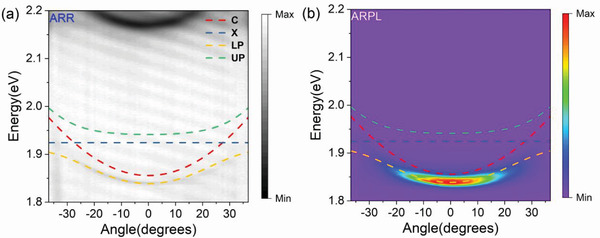
Angle‐resolved a) reflectivity and b) emission spectra of the nanoplatelet microcavity. C, cavity dispersion; X, hh‐e excitons; LP/UP, upper/lower polariton branch. The green and yellow dashed lines represent the theoretical fitting curves of the UP and LP dispersion. The blue dashed line represents the uncoupled nanoplatelets' hh‐e excitons, and the red dashed line represents the cavity fitted from a coupled harmonic oscillator model.

The polariton dispersion properties were measured by home‐built angle‐resolved (equivalent to a function of in‐plane wavevector *k*_∥_) reflectivity (ARR) and photoluminescence (ARPL) spectra using a Fourier imaging configuration (see Figure S5, Supporting Information) at room temperature (300K). Due to the limited lifetime of the cavity mode, the photon leaking from the microcavity carries the same energy and momentum as the polariton, which makes it possible to simultaneously measure the angle and energy of the polariton with this method. **Figure** **2**a,b shows the *k*_∥_‐space mapping of the ARR and ARPL spectra, respectively. The dispersion relationship obtained in the ARPL mapping agrees well with that in the ARR measurement, except for small difference in energy attributed to the slightly different pump spot location. In addition, we did not observe any emission from the uncoupled hh‐e exciton located at 1.904 eV. The upper polariton (UP) branch dispersion is barely visible in ARR and ARPL mapping, which is typical for microcavities with large Rabi splitting energy. This is mainly attributed to the continuous absorption band of the electron‐hole in high energy region, thermal relaxation, and ultrashort lifetime of UP arising from the intense interaction between polariton and polariton reservoir.^[^ ^19^ ^]^ Nonetheless, the dispersion of the lower polariton (LP) branch is consistent with the strong exciton‐photon coupling regime: 1) the dispersion curve curvature decreases at large angles; 2) an obvious inflection point is distinguished when approaching the exciton energy near ±25°, which results from the anti‐crossing behavior of the strong coupling between the microcavity photons and nanoplatelet hh‐e excitons. The UP, LP, and cavity mode dispersion curves were fitted according to the coupled harmonic oscillator model (see Methods S2, Supporting Information). Through fitting, a Rabi splitting energy of 2Ω = 76 meV and a negative exciton‐photon detuning of Δ = − 68 meV were obtained. The Rabi splitting energy satisfies the relationship of

(1) $$2\Omega >\left(\gamma_{cav}+\gamma_{EX}\right)/2$$

where *γ*_*cav*_ = 27 meV and *γ*_*EX*_ = 64 meV are the cavity and exciton linewidth, respectively. This indicates that the microcavity works in the strong coupling regime.^[^ ^20^ ^]^ For comparison, the microcavity ARR spectrum was calculated according to the transfer matrix method (TMM), as shown in Figure S6 (Supporting Information). It should be noted that the transfer matrix model is an approximation without considering the number of nanoplatelets, which results in smaller Rabi splitting energy (44 meV) than experiment. The Hopfield coefficients, which theoretically describe the contributions from exciton and photon components in a polariton branch and represent how the polaritons are hybridized by excitons and photons,^[^ ^21^ ^]^ at different detection angles of LP are shown in Figure S7 (Supporting Information). LP behaves in a more photon‐like state with an extremely small effective mass, fast decay lifetime in small angles, and a more exciton‐like state at large angles.

### 2.3 Polariton BEC and Lasing

**Figure 3:**
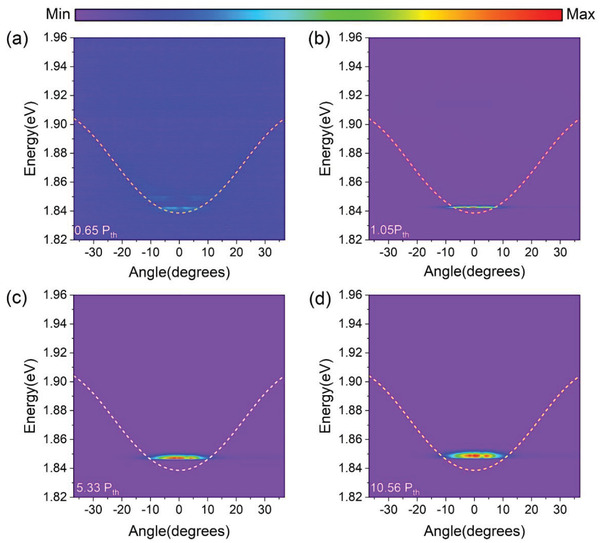
Pump power‐dependent ARPL mapping. The yellow lines are the LP dispersion curves. a) Measured at 0.75 P_th_. LP emission shows a broad distribution at all angles. The mapping shows strong emission at several angles due to the existence of spatial traps. b) Measured at 1.05 P_th._ The emission spectrum concentrated near 0 degrees at the ground state, indicating the onset of polariton condensation. c) Measured at 5.33 P_th_. The ground state is massively occupied, and the peak is strongly blue‐shifted and broadened. d) Measured at 10.56 P_th_. The emission is further blue‐shifted and broadened.

To reach the nonlinear regime, the system was off‐resonantly pumped with a femtosecond laser (pulse duration = 150 fs) centered at 400 nm and a spot size of 30 µm in radius. **Figure** **3**a–d shows the contour maps of representative ARPL with pump power of 0.65, 1.05, 5.33, and 10.56 of the pump threshold (*P*_th_ = 0.5 µJ cm^–2^), respectively. At a low pump power of 0.65 P_th_, the LP dispersion exhibits a broad emission at several angles, which may result from the spatial traps formed by nanoplatelet self‐stacking.^[^ ^22^ ^]^ When the pump power reached 1.05 P_th_, the polariton dispersion mapping near 0 degrees exhibited a much stronger emission along with a sharp increase in intensity and a decrease in linewidth, indicating the onset of polariton BEC and lasing. Under higher pump powers of 5.33 and 10.56 P_th_, the ground states near the minimum of the LP dispersion (1.839 eV) are massively occupied without emissions at other angles, which is significantly different from the conventional photonic laser. In the latter, the emission should massively occupy the minimum of the cavity photonic dispersion (1.856 eV).^[^ ^4^, ^8^ ^]^ The spontaneous macroscopic occupation of the LP ground state is the strong evidence for polariton condensation and lasing. The polariton laser stability was measured by continuous recording at 3 P_th_ showing in Figure S8 (Supporting Information). The output laser intensity can retain 90% of its original intensity after 3 hours (1.08 × 10^7^ shots), proving the outstanding photostability of nanoplatelets in the microcavity.

**Figure 4:**
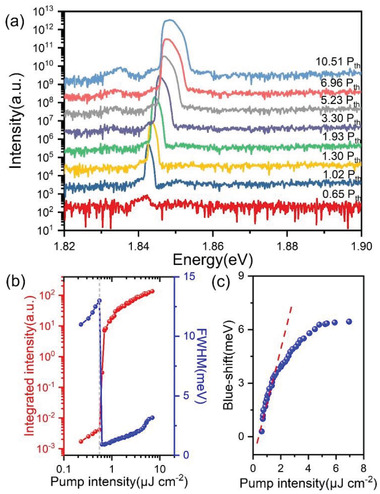
Nanoplatelet microcavity exciton‐polariton BEC properties. a) Ground‐state PL spectra as a function of pump power. The curves were shifted along the longitudinal axis for clarity. Semilogarithmic graphs were used to show the evolution of the spectrum more intuitively. A rapid increase in emission intensity and a line width narrowing occurs at 1.02 P_th_, indicating the onset of polariton BEC. The broadening and blueshift of the emission spectra can be well‐observed as the pump power increases. b) Pump power‐dependent ground‐state emission intensity(red) and FWHM (blue). The grey dashed line marks the threshold of 0.5 µJ cm^–2^, where the FWHM narrows and intensity increases dramatically. c) The ground‐state emission peak blueshift as a function of pump power. The polariton‐polariton interaction leads to a linear trend (red dashed line) at the beginning of condensation.

In **Figure** **4**a, the LP ground‐state emission spectra measured at 0 degrees as a function of pump power is shown in a logarithmic scale. Upon reaching the pump threshold, the ground state emission intensity experiences a sharp increase, accompanied by FWHM narrowing and blueshift of the peak. This indicates a transition from the linear regime to the nonlinear regime and polariton lasing. We plotted the integrated emission intensity of the LP ground state and pump intensity in a log–log scale (L–L graph, Figure 4b, red line) to quantify the polariton nonlinear behavior. After reaching the pump threshold of 0.5 µJ cm^–2^, a sharp increase of approximately four orders of magnitude in emission intensity is observed. In addition, the FWHM of the ground state emission is shown as the blue‐line in Figure 4b, with a slight increase before condensation (≈10 meV – ≈13 meV) due to the polariton‐polariton reservoir interaction. Then, the linewidth drastically narrows to 0.93 meV upon reaching the threshold, suggesting a strong increase in temporal coherence. The linewidth broadens again as the pump power increases, which is attributed to the polaritons interaction with excitons, phonons, and polaritons through their exciton component.^[^ ^23^ ^]^ The pump‐power‐dependent emission intensity and linewidth unambiguously indicate polariton‐polariton stimulated scattering and condensation. Since the ratio of photon components of the LP ground‐states is as high as 0.83, condensation is still observable at a pump power of ≈13 P_th_. The pump power‐dependent ground‐state emission energy blueshift above the condensation threshold is plotted in Figure 4c. A maximum blueshift of 9 meV of the polariton condensate is observed, which is much smaller than the detuning of 68 meV. At the beginning of condensation, the polariton‐polariton scattering leads to a linear growth in the ground‐state energy.^[^ ^11b^ ^]^ The blueshift of the ground state endures a sublinear increase as the pump power is further increased, which is common in polariton condensation owing to polariton density saturation.^[^ ^7^, ^8^ ^]^

### 2.4 Spatial and Temporal Coherence

**Figure 5:**
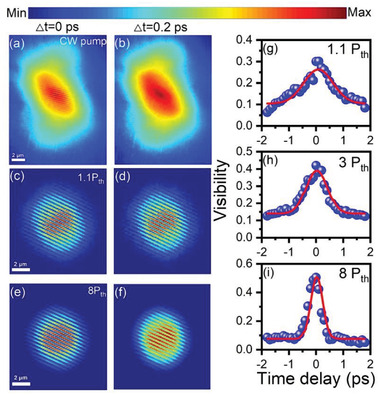
Representative temporal and spatial coherence of polariton BEC measured using a home‐built Michelson interferometer. The pseudocolor pictures in the first and second rows are the interference patterns measured at time delays between the interferometer's two arms of 0 ps and 0.2 ps, respectively. Scale bar: 2 µm. a,b) CW 532 nm pump, below the threshold; c,d) 1.1 P_th_, e,f) 8 P_th_. g,i) Visibility as a function of time delay with a pump power of 1.1, 3, and 8 P_th_. The experimental data (blue dots) are fitted with Gaussian functions (red lines).

Since polariton condensation is derived from bosonic final‐state stimulation and ground‐state coherent macroscopic occupation, additional significant and strong evidence of polariton lasing is manifested by an increase in temporal coherence and build‐up of long‐range spatial coherence.^[^ ^24^ ^]^ The coherence property of polariton BEC was measured using a home‐built Michelson interferometer (Figure S9, Supporting Information), where the time delay(*τ*) between the two arms is controlled by the delay line. With this configuration, the coherence property and real‐space image can be simultaneously measured. Figures S10 and S11 (Supporting Information) show real‐space images measured from one arm of the Michelson interferometer below and above the condensation threshold, respectively. In the linear regime, the emission image almost fills the entire picture. When the pump power reached the BEC threshold, the emission collapses to a circular spot with a radius of ≈3 µm, which is a prominent feature of polariton condensation and lasing.^[^ ^2^, ^25^ ^]^ The representative intensity‐dependent interference images measured at *τ* = 0 ps and 0.2 ps are shown in **Figure** **5**a–f and Figure S12 (Supporting Information). Below the condensation threshold, the polariton–polariton interaction is considerably low and polariton can be treated as ideal Bose gas. The particle size of polariton is their thermal de Broglie wavelength,^[^ ^26^ ^]^ therefore, the polariton should have characteristic coherence length in the order of their thermal de Broglie wavelength. A flat interference pattern with low contrast can be observed in the range of 1.44 µm in Figure 5a. Nevertheless, above the threshold, the unambiguous interference fringes are readily identified along with a distance as long as 7.6 µm. The interference fringe intensity across the autocorrelation point is plotted in Figure S13 (Supporting Information) to give quantitative comparison. This rapid build‐up long‐range spatial coherence is strong evidence for realizing polariton BEC in the nanoplatelet microcavity. At *τ* = 0.2 ps, the interference fringes are still visible above the threshold but almost invisible below the threshold. The polariton‐polariton interaction below the condensation threshold is weak therefore the dephasing time is considerably short, which is relatively shorter than the time delay (0.2 ps) in our experiment. The measurement results of interference fringes directly indicate that the temporal and spatial coherence of the nanoplatelets microcavity system increases quickly after crossing the condensation threshold.

In the next step, we analyzed the representative temporal coherence of the microcavity above the threshold, as shown in Figure 5g–i. At *τ* = 0 ps, the interference patterns show excellent visibility up to 0.3, where the visibility is defined as

(2) $$V\;=\;\left(I_{max}-I_{min}\right)/\left(I_{max}+I_{min}\right)$$

*I*_*max*(*min*)_ is the maximum (minimum) intensity of the interference fringes. The extracted visibility as a function of delay between the two arms can be fitted using a Gaussian function,^[^ ^27^ ^]^ and a coherence time of 1.26 ps is derived for 1.1 P_th_. This estimated coherence time is comparable to other room‐temperature polariton lasing using inorganic materials.^[^ ^8^, ^24^, ^28^ ^]^ When the pump power is 8 P_th_, the coherence time of the system decreases to 0.52 ps. The decrease in the system temporal coherence mainly caused by polariton‐polariton scattering, which leads to phase decoherence.^[^ ^30^ ^]^

### 2.5 Polariton BEC Dynamics

**Figure 6:**
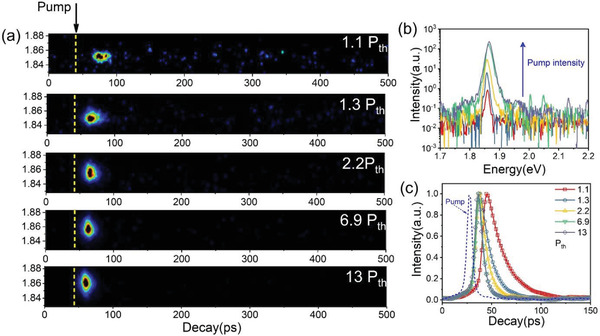
Representative ground‐state emission dynamics as a function of the pump power measured by a high‐temporal resolution streak camera system. a) Streak camera image. The yellow dashed lines are the position of the corresponding pump pulse. b) PL spectra extracted from the corresponding streak camera image. The curves were translated in the longitudinal direction for intuitive observation. c) Dynamics of the ground‐state emission. The blue dashed line represents the IRF measured by the pump pulse. The dot‐line curves were obtained by deconvoluting the experimental data and the IRF.

In the following step, the ground‐state of the polariton dynamics was measured to study the polariton–polariton interaction. Since the LP emission below the condensation threshold excited by the femtosecond laser is considerably weak, here, the lowest power used is 1.1 P_th_. The pump power‐dependent streak camera images are shown in **Figure** **6**a. A rising edge between the ground‐state emission and the pump laser can be well observed. As the pump power increases, the rising edge becomes shorter, which unambiguously proves that polariton–polariton scattering effectively shortens the accumulation time to reach the lasing threshold.^[^ ^6^ ^]^ The transient emission spectra in the 0–200 ps delay window are shown in Figure 6b. Despite the poor spectral resolution of the streak camera system, the blueshift and broadening of the ground‐state emission can still be distinguished. The ground‐state emission dynamics extracted from the corresponding streak camera images is shown in Figure S14 (Supporting Information). The instrument response curve (IRF, the blue line inFigure 6c) is used to perform deconvolution calculation on the measured curve to analyze the dynamic process more accurately. Those curves can be fitted by a single‐exponential function. The lifetime constant at 1.1 P_th_ is estimated to be 18 ps. When the pump power increases to 2.2 P_th_, the lifetime sharply decreases to 5.8 ps, which was close to the streak camera system resolution limit. The sharply shortened rising edge and decreased emission lifetime are additional distinct evidence for the realization of BEC and polariton lasing in the nanoplatelet microcavity system.

## 3 Conclusion

In conclusion, we have demonstrated room‐temperature polariton BEC in a colloidal CdSe/CdS nanoplatelet‐based microcavity for the first time with a large Rabi splitting energy of 76 meV. The polariton BEC and lasing at room temperature in this system is unambiguously demonstrated through pump‐power dependent superlinear ground‐state emission, blueshift, and broadening of the linewidth, long‐range build‐up spatial and temporal coherence upon reaching the threshold. The successful realization of the stable polariton lasing in this low‐Q microcavity, high exciton binding energy, and ease of fabrication considering the colloidal solution preparation process significantly reduce the physical requirements for realizing room‐temperature polariton BEC. Furthermore, our findings discover and open up a new platform of great significance, which provides an alternative choice for realizing large‐area, low‐cost, high‐performance polariton devices with colloidal nanoplatelets.

## 4 Experimental Section

### CdSe/CdS Nanoplatelets Synthesis

The 4MLs CdSe nanoplatelet core were prepared by reacting Cadmium(myristate)_2_ with selenium powder in 1‐octadecene at 240 °C following the literature.^[^ ^12a^ ^]^ The CdS shell was grew on the CdSe core using a hot‐injection method.^[^ ^30^ ^]^ Details are given in the Supporting Information.

### Microcavity Fabrication

The bottom DBR was composed of 12 alternating pairs of silicon dioxide (SiO_2_, n = 1.45) and titanium dioxide (TiO_2_, n = 2.25) on a commercial quartz wafer. High‐purity materials were used to prepare the DBR using an e‐beam evaporator at high vacuum. The relatively thick nanoplatelet film was then deposited on the bottom DBR by drop‐casting a concentrated solution (50 mgml^‐1^, volume ratio, octane: hexane = 1: 9). The drop‐casting method provides a relatively flat surface, as shown in the AFM image in Figure S3 (Supporting Information). The top DBR composed of eight alternating pairs of calcium fluoride (CaF_2_, *n* = 1.23) and zinc sulfide (ZnS, *n* = 2.3) was deposited on top of the nanoplatelet film by an e‐beam evaporator at relatively low temperature (<50 °C) to prevent nanoplatelet bleaching. Pictures of the nanoplatelets film deposition on the bottom DBR and the whole cavity are shown in Figure S4 (Supporting Information).

### Characterization

The TEM and HRTEM images were measured by a JOEL company JEM‐2100 TEM, with an accelerating voltage of 200 kV. The absorption spectrum was measured with a Shimadzu UV 3600 UV–vis spectrophotometer, and a quartz cuvette with a light path of 1 cm was used as the sample cell. The PL spectrum was measured with Edinburgh FLS900. The refractive index of the nanoplatelet film was measured using a J.A.Woollam company M‐2000 ellipsometer.

Angle‐resolved photoluminescence and reflectivity spectra were measured in a home‐built micro photoluminescence setup within Fourier imaging configuration. The detailed experimental setup is depicted in Figure S5 (Supporting Information). A high numerical aperture 50× microscope objective (Nikon TU Plan ELWD, N.A. = 0.6) was used to cover the angle range of ±36.8°. In the linear regime, a continuous 532 nm laser was used to measure the LP photoluminescence. To explore the nonlinear regime, a 400 nm laser pulse was generated by Coherent's Legend‐F‐1k femtosecond laser (800 nm, 150 fs, 1 kHz) through a barium metaborate (BBO) crystal was used as the pump source. For the reflectivity measurement, a Xenon lamp was used. The spatial and temporal coherence properties were measured with home‐built Michelson interferometer, as shown in Figure S9 (Supporting Information). The decay kinetics of the LP ground‐state are measured by a Hamamatsu C5680 streak camera working in the synchronously scanned mode. The time range and spectral resolution are 800 ps and 1.2 nm, respectively, with the corresponding FWHM of the instrument response function of ≈4 ps.

### Statistical Analysis

The emission spectra were fitted with the Lorenz equation to obtain the peak and FWHM. ARR mapping was calculated according to the incident Xenon lamp spectrum. The emission intensity was normalized according to the integrated time of the measurement. The emission/reflection angle(*θ*_*i*_) was calculated according to the pixel index(*i*) of the spectrapro CCD, where *θ*_*i*_ = tan^−1^(*C* × *i*), C = tan*β*/*n*, *n* is the total detectable pixels of the back focal plane on the spectrapro CCD, *β* = 36.8° corresponding to the objective detectable angle, and C is the scale factor. The original grayscale intereference pictures taken from CCD were plotted with MATLAB to obtain pseudo color images and the intensities were obtained with ImageJ software. The scale bar was calibrated with microscope graticules following a standard process. The intereference fringer visibilities were calculated with an area of 40 × 40 pixels around the autocorrelation point. The decay curve deconvolution process was performed with DelayFit software.

## Conflict of Interest

The authors declare no conflict of interest.

## Supporting information

Supporting Information

## Data Availability

The data that support the findings of this study are available on request from the corresponding author. The data are not publicly available due to privacy or ethical restrictions.
