# Supplementary material for: Ultralow Threshold Room Temperature Polariton Condensation in Colloidal CdSe/CdS Core/Shell Nanoplatelets
Source: Adv Sci (Weinh). 2022 Apr 24;9(18):2200395. doi: 10.1002/advs.202200395 (PMC9218774; doi:10.1002/advs.202200395)
Supplement: Supplementary file 1 — Supporting Information [file ADVS-9-2200395-s001.pdf]

ADVANCED SCIENCE
Open Access

Supporting Information

for *Adv. Sci.*, DOI 10.1002/advs.202200395

Ultralow Threshold Room Temperature Polariton Condensation in Colloidal CdSe/CdS Core/Shell Nanoplatelets

Hongyu Yang, Lei Zhang, Wenbin Xiang, Changgui Lu, Yiping Cui and Jiayu Zhang**

Supporting Information

Ultralow threshold room temperature polariton Bose-Einstein condensation in colloidal CdSe/CdS core/shell nanoplatelets

Hongyu Yang, Lei Zhang, Wenbin Xiang, Changgui Lu, Yiping Cui, Jiayu Zhang**

Dr. H. Yang, Dr. L. Zhang, Dr. W. Xiang, Prof. C. Lu, Prof. Y. Cui and Prof. J. Zhang
Advanced Photonics Center, Southeast University, Nanjing 210096, Jiangsu, China

Dr. L. Zhang
School of Sciences, Nantong University, Nantong 226019, China

*E-mail: changguilu@seu.edu.cn; jyzhang@seu.edu.cn

Method S1. Synthesis of CdSe/CdS core/shell nanoplatelets

Chemical used. Cadmium oxide (CdO, >99.99%), cadmium nitrate tetrahydrate ($Cd(NO_3)_2{\cdot}4H_2O$,98%), sodium myristate (Namyr>99%), cadmium acetate dihydrate ($Cd(Ac)_2{\cdot}2H_2O$, >99.99%, trace metals basis), selenium powder (Se,100 mesh, 99%), 1-octadecene (ODE,90%, technical grade), oleic acid (OA, 90%), oleylamine (OAm, 70%), and octanethiol (OT, >98.5%) were purchased from Sigma-Aldrich. All reagents were used directly without further purification.

Preparation of Cadmium Myristate. Cadmium myristate ($Cd(myr)_2$)was synthesized using previously published method[1]. First, 3.13 g of Namyr was dissolved in 250 mL of methanol overnight, and 1.23 g of $Cd(NO_3)_2{\cdot}4H_2O$ was dissolved in 40 mL of methanol. When the two solutions are completely dissolved, the two solutions are mixed and stirred for approximately 1 hour, and a large amount of white flocci can be seen. After that, the flocci were filtered through a Buchner funnel. The obtained white precipitate was washed at least three times with methanol. The finally obtained precipitate was dried overnight in a vacuum environment and then stored at room temperature.

Preparation of the shell growth citation precursor (Cadmium Oleate,$Cd(OA)_2$). CdO(4 mmol), OA(8 mmol) and ODE(40 mL) were added to a 150 mL three-necked flask. Under magnetic stirring, we used a Schlenk-line to remove water and oxygen in vacuum at 120℃. Then the solution was heated to 240 ℃ under Ar atmosphere until the solution was clear. After cooling, the solution was stored in a 40 mL chromatography bottle.

Synthesis of 4-MLs-thick CdSe NPLs. The core was synthesized following the methods from S. Ithurria[2]. This thick core has a regular shape and no defect-related emission can be observed. In general, 340 mg Cd(myr)$_2$, 24 mg Se and 30 mL ODE were added in a 150 mL three-necked flask. Under magnetic stirring, we used a Schlenk-line to remove water and oxygen in vacuum at 90 °C for at least an hour. The solution was then heated to 240°C under Ar atmosphere. At 195 °C, 160 mg Cd(Ac)$_2$·2H$_2$O was added to promote the formation of 2D nanoplatelets. The reaction was carried out at 240 °C for about 10 minutes and then rapidly cooling to room temperature. During the cooling, 1 mLof OA was added at 180 °C. The nanoplatelets were precipitated from the crude solution using hexane and ethanol and finally dissolved in hexane. The solution is kept in dark until needed.

CdS shell growth. The shell is deposited on the core using a hot-injection method[3]. An appropriate amount of nanoplatelet core solution,3 mL ODE and 2 mL Cd(OA)$_2$ were added into a 50 ml three-necked flask. Under magnetic stirring, we used a Schlenk-line to remove water, hexane and oxygen in vacuum at 80 °C for at least an hour. Then the temperature was quickly raised to 300°C, and 1 mL of pre-degassed OAm was injected at 180 °C. In the meantime, 6 mL anion precursor (prepared using 252 µL OT and 36 mL ODE) was injected using a syringe pump at 3 mL/h. The solution was annealed at 300°C when finishing the injection for 10 minutes and then rapidly cooling to room temperature. 1 mL OA was injected at 190 °C and then 5 mL of hexane was injected at 70 °C. The core/shell nanoplatelets were precipitated by size-selective precipitation method and dissolved in hexane.

Method S2. Coupled harmonic oscillator model

The eigen equation of the exciton-polariton mixed state can be considered as the linear superposition of the wavefunctions of excitons and photons with the same wavevector. In the coupled harmonic oscillator model, the diagonalized Hamiltonian $\widehat{\mathcal{H}}$ and the eigenvalue $E_{XP}$of the system satisfy the following stationary Schrödinger equation[4]:

$$\widehat{\mathcal{H}}|\psi\rangle = \begin{pmatrix} E_C & \Omega \\ \Omega & E_X \end{pmatrix}\begin{pmatrix} C \\ X \end{pmatrix} = E_{XP}\begin{pmatrix} C \\ X \end{pmatrix}$$

where, E_C, E_X, E_{XP} are the eigen energy of microcavity photon, exciton and polariton, respectively, $|C|^2$ and $|X|^2$ are the Hopfield coefficients and Ω is the exciton–photon interaction strength. Therefore, the eigenvalue of the polariton is

$$E_{LP,UP}(\theta) = \frac{1}{2}\left[E_C(\theta) + E_X(\theta) \mp \sqrt{(2\Omega)^2 + \Delta^2}\right]$$

where, $E_C(\theta) = E_c(0)/\sqrt{\left(1-\left(\frac{\sin(\theta)}{n_{eff}}\right)^2\right)}$ is the dispersion relationship of the microcavity photon, n_{eff} is the effective reflective index of the microcavity, $E_X(\theta)$ is fixed at 1.924 eV and $\Delta = E_C - E_X$ is the detuning between exciton and photon.

The effective refractive index can be calculated according to the following equations:

$$n_{eff} = \frac{n_{NPL}L_{NPL} + n_{Bragg\_b}L_{Bragg\_b} + n_{Bragg\_t}L_{Bragg\_t}}{L_{NPL} + L_{Bragg\_b} + L_{Bragg\_t}}$$

$$L_{Bragg\_b(t)} = \frac{\lambda_0}{2}\frac{n_{1\_b(t)}n_{2\_b(t)}}{n_{NPL}(n_{2_{b(t)}} - n_{1_{b(t)}})}$$

$$n_{Bragg\_b(t)} = \frac{n_{1\_b(t)}L_{1\_b(t)} + n_{2\_b(t)}L_{2\_b(t)}}{L_{1\_b(t)} + L_{2\_b(t)}}$$

Where, $n_{NPL}, n_{1\_b(t)}, n_{2\_b(t)}$ are the refractive index of the nanoplatelet film, the alternating bottom(top) Bragg reflector material, $L_{NPL} = 1\ \mu m$ is the nanoplatelet film thickness, $n_{Bragg\_b(t)}$ is the bottom(top) Bragg reflector effective refractive index, $L_{Bragg\_b(t)}$ is the bottom(top) Bragg reflector penetration length and $\lambda_0 = 650\ nm$ is the Bragg reflector resonance wavelength. Through fitting, $n_{eff} = 1.75$ is calculated.

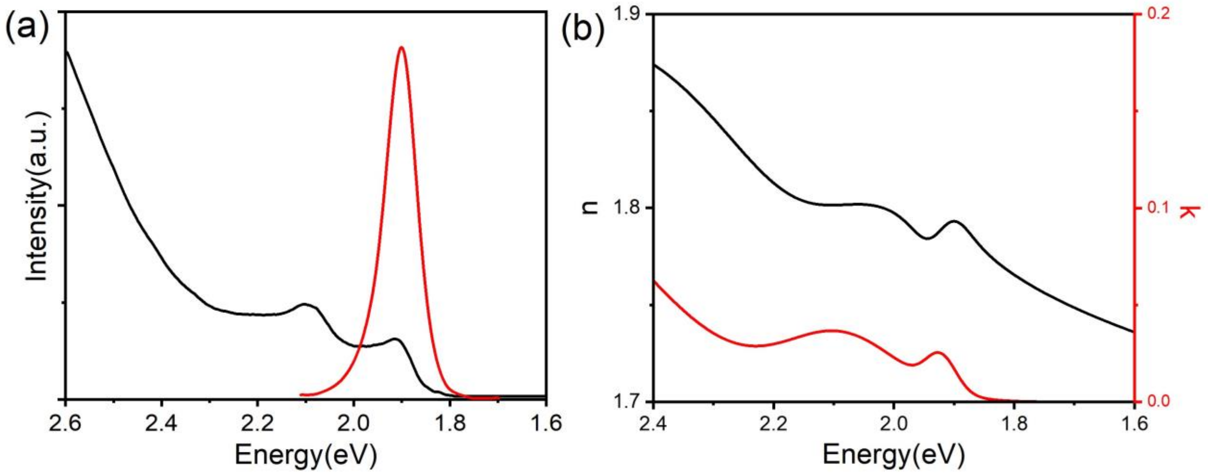

Figure S1. Characteristics of the CdSe/CdS nanoplatelet film. (a)Absorption (black line) and PL (red line) spectra; (b) real (black line) and imaginary (red line) parts of the refractive index.

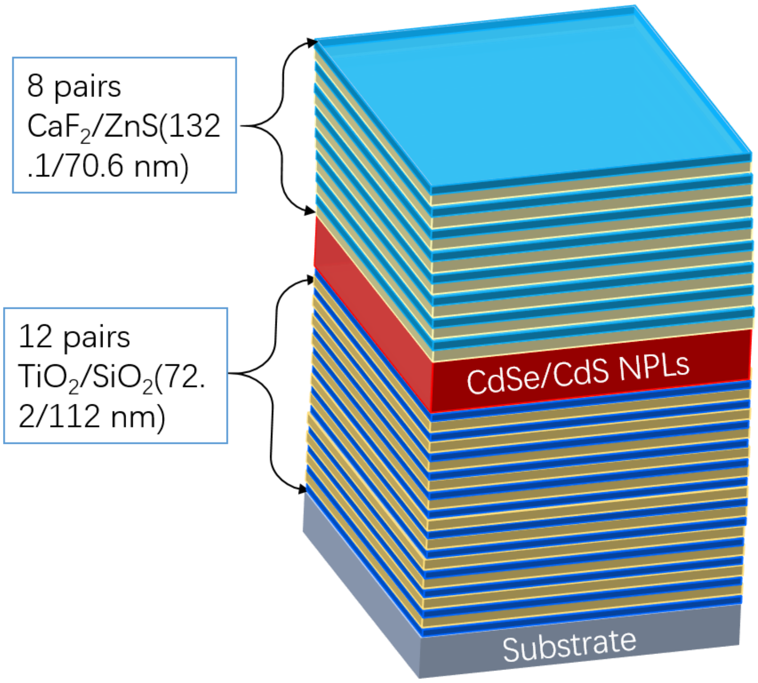

Figure S2. Schematic diagram of the microcavity.

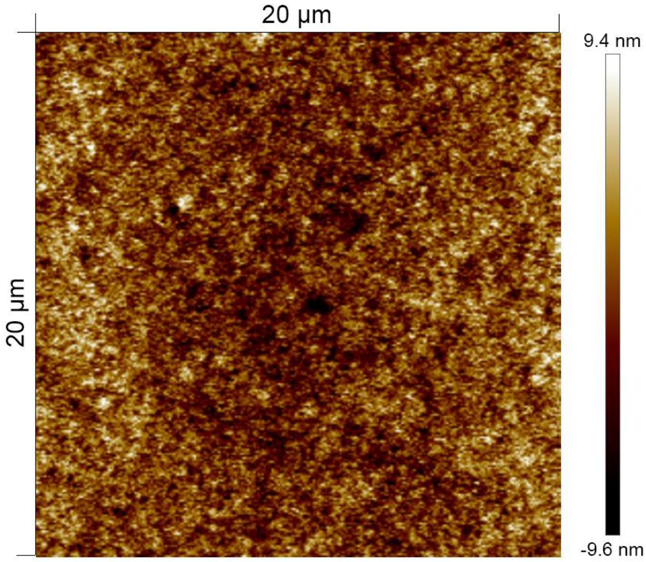

Figure S3. AFM image of the nanoplatelet film deposited on the bottom DBR. The root-mean square roughness is ~2.72 nm.

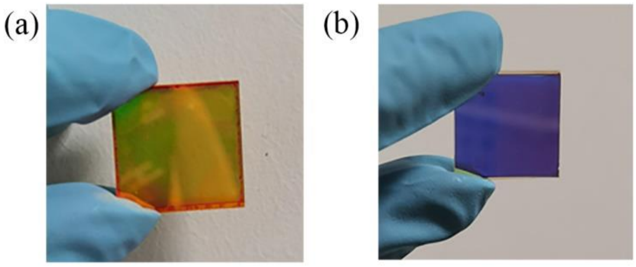

Figure S4. Picture of (a) nanoplatelet film deposited on the bottom DBR (b) whole cavity.

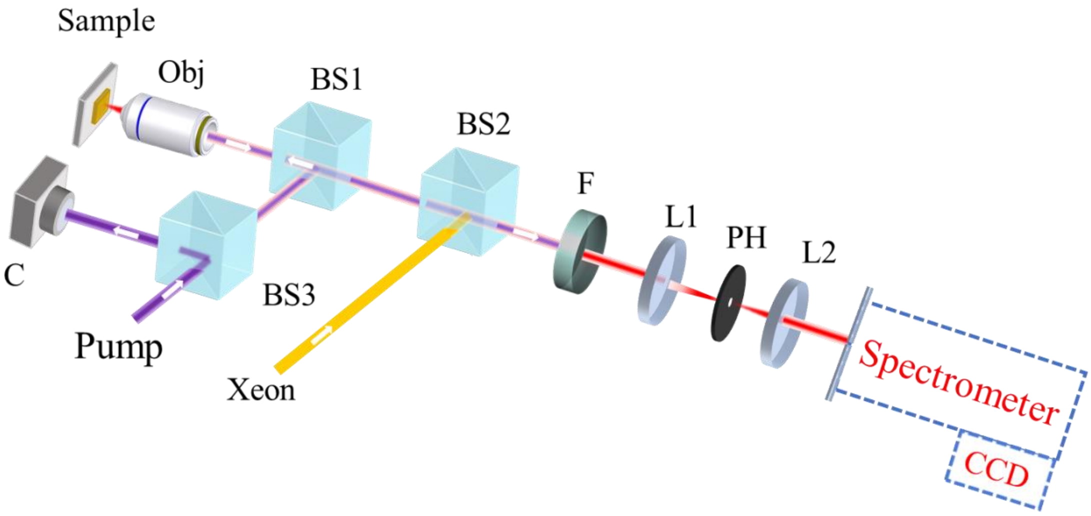

Figure S5. Home-built angle-resolved reflectivity/photoluminescence based on Fourier imaging configuration. The sample is fixed on a three-dimensional translation stage, and the reflection and emission signals of the sample are pumped and collected through the same objective lens. For emission measurement, the pump light is a linearly polarized 532 nm continuous laser diode (25 μW) and a 400 nm femtosecond pulsed laser generated by a Coherent Legend-1F (800 nm, 1 kHz, 150 fs) which is frequency-doubled with a β-barium metaborate crystal(β-BBO). The pump power was adjusted by a continuously variable neutral filter and measured by power energy meter (Thorlabs, PM100D) with a silicon power head (Thorlabs, S120VC). For reflection, we use xenon lamp as the illumination source. The signal collected by the objective lens is focused through lens L1 on the focal plane, where a diaphragm is used so that only the signal near the pump light can pass. The k-space image was generated by using lens L2 according to Fourier transformation and then projected on the slit of a spectrometer equipped with a CCD. The energy, intensity and momentum information of the emitted photons can be directly obtained through this setup. Obj: Nikon TU Plan EWLD 50 ×, N.A.=0.6, corresponding to the collection angle= ±36.8°. BS1, BS2 and BS3 are the nonpolar beam splitters with splitting ratio of 50:50(Thorlabs, BS013). L1 and L2 are lenses with focal length of 20 cm and 10 cm, respectively. PH: diaphragm. C: A Nikon DS-Qi1Mc Peltier cooling CCD camera. The spectrometer is an Acton Spectrapro 300i Cherny-Turner imaging spectrometer with a focal length of 300 mm with a Hamamatsu 128*1024 array CCD work at -30℃. For Figure 2, a grating with 300 lines/mm was used, which corresponding to the resolution of 0.26 nm. For Figure 3, a grating with 1200 lines/mm was used, which corresponds to the resolution of 0.05 nm. The spectrometer was calibrated before operation.

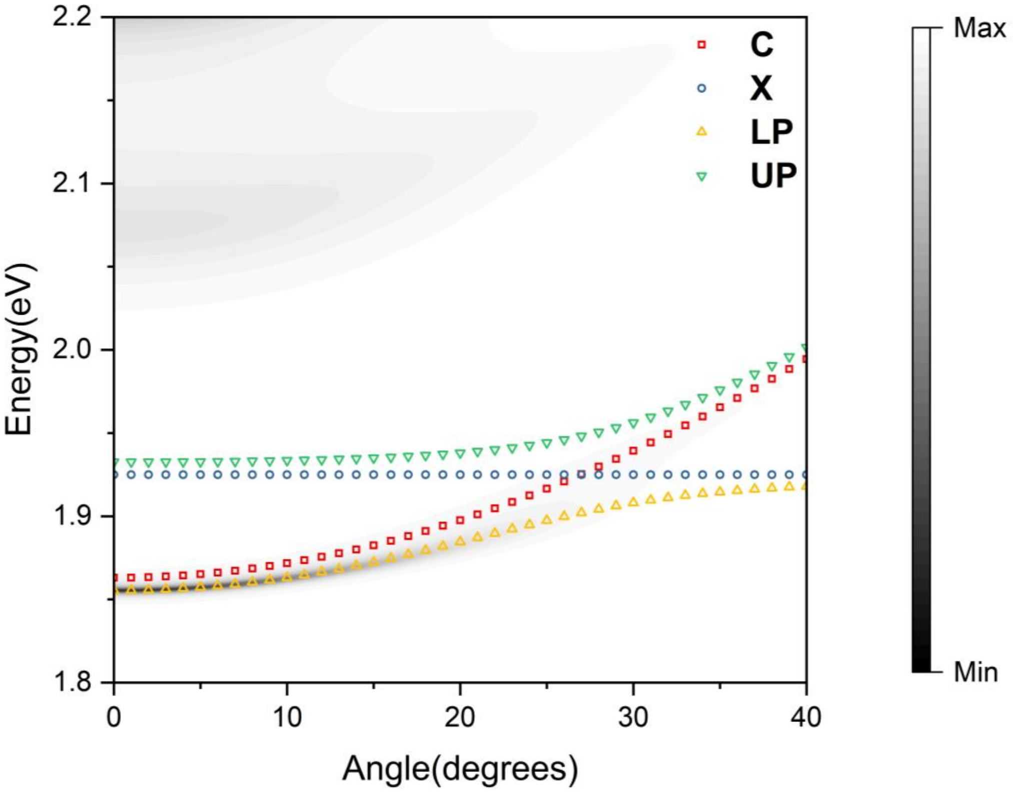

Figure S6. Microcavity ARR mapping calculated according to TMM methods. The dots are the fitting according to the coupled harmonic oscillator model.

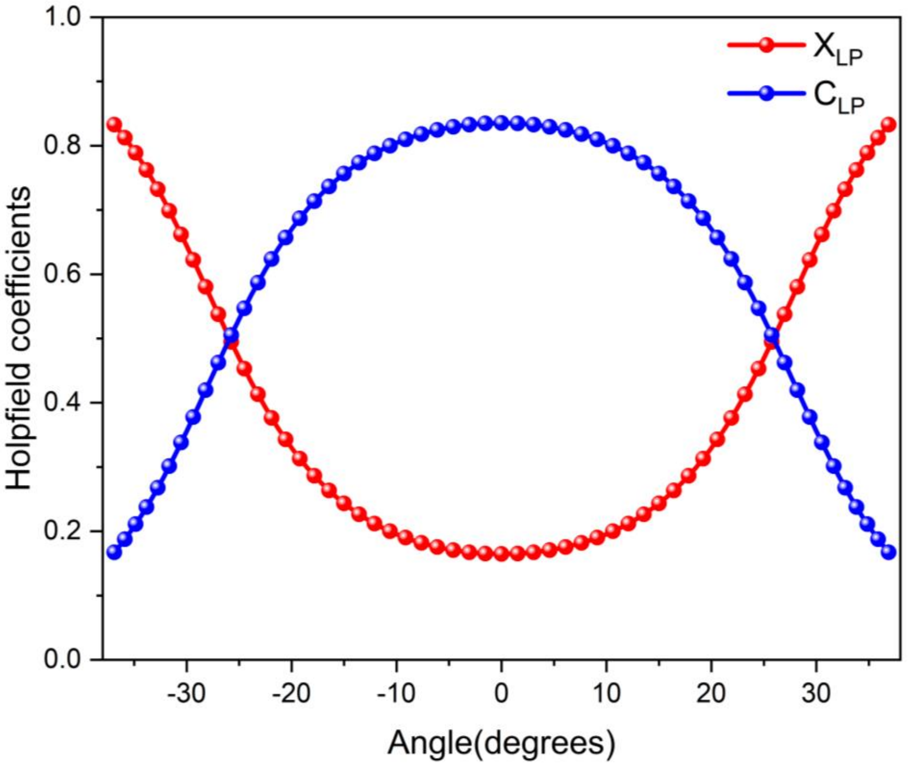

Figure S7. The photon/exciton ratio of the polariton as a function of the angle, that is, the Holpfield coefficient. At low angles (-10°–10°), the photon component is significantly larger than the exciton component.

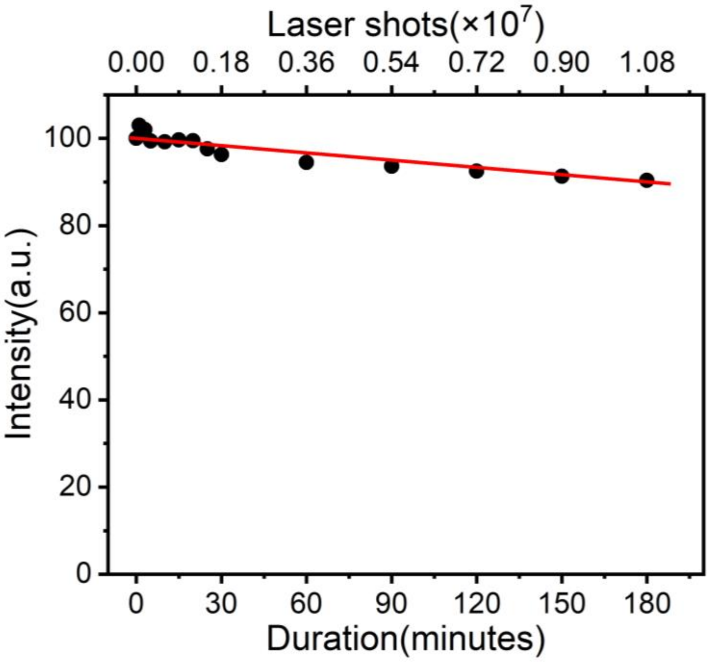

Figure S8. Polariton laser intensity monitored as a function of the continuous excitation duration (pump intensity: 3Pth). The red line is a fitted line to the data. There was a 10% drop in the intensity after 3 h of continuous operation ($1.08{\times}10^7$ shots).

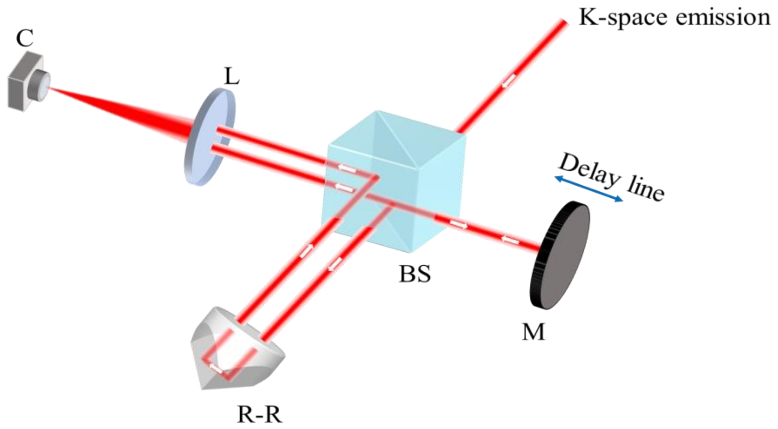

Figure S9. Home-built Michelson interferometer. The *k*-space image emitted from L2 in Figure S1 was collimated to the nonpolarizing beam splitter (BS) and was divided into two paths. One path of transmitted light is reversed by a retroreflector (RR, Thorlabs, PS975), and the other path of the reflected light is a mirror fixed on the electric translation stage, which reflects the image along the original path. The position of the delay line (Thorlabs, MTS25/M-Z8) is controlled by a computer to create a time difference between the two arms of the interferometer. The light emitted by the two arms of the interferometer refocuses the k-space image to the real space through lens L (focal length of 20 cm), and is collected and measured by the CCD camera.

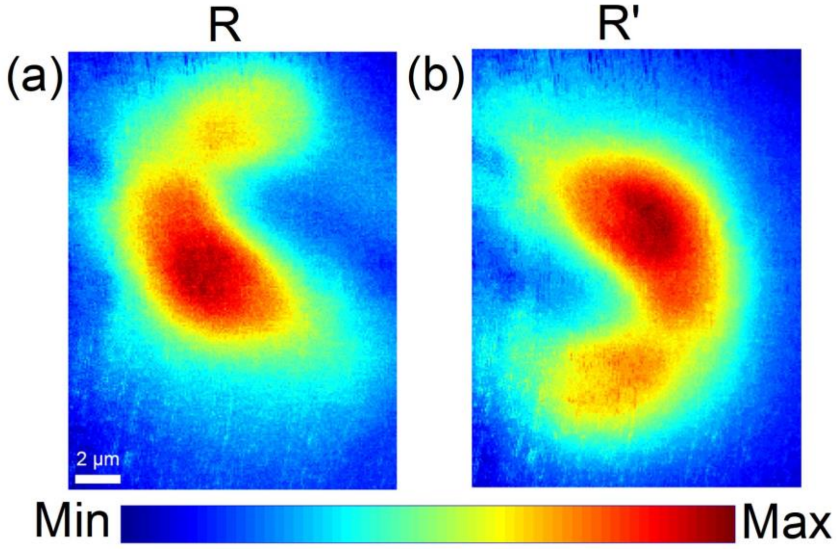

Figure S10. (a) (b) Images of the two arms of the Michelson interferometer when the microcavity was pumped by 532 nm continuous light. The two images are clearly centrally symmetrical. Scale bar: 2 μm.

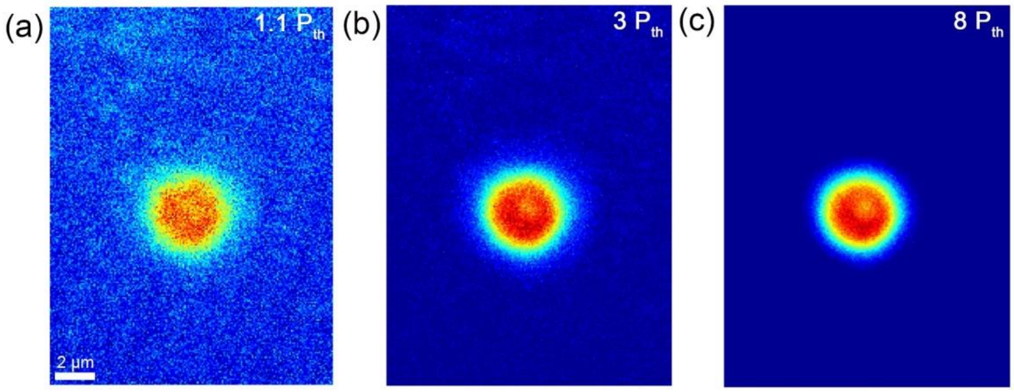

Figure S11. The real-space image measured on one arm of the Michelson interferometer above the condensation threshold. The image shows a round spot with a radius of approximate 3 μm. Scale bar: 2 μm. (a) 1.1 P_{th} (b) 3 P_{th} (c) 8 P_{th}.

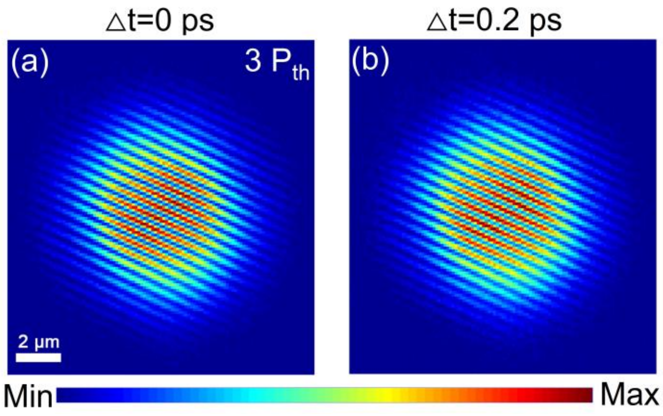

Figure S12. Interference pattern measured at 3 P_{th}. Time delay between the interferometer's two arms of (a) 0 ps and (b) 0.2 ps.

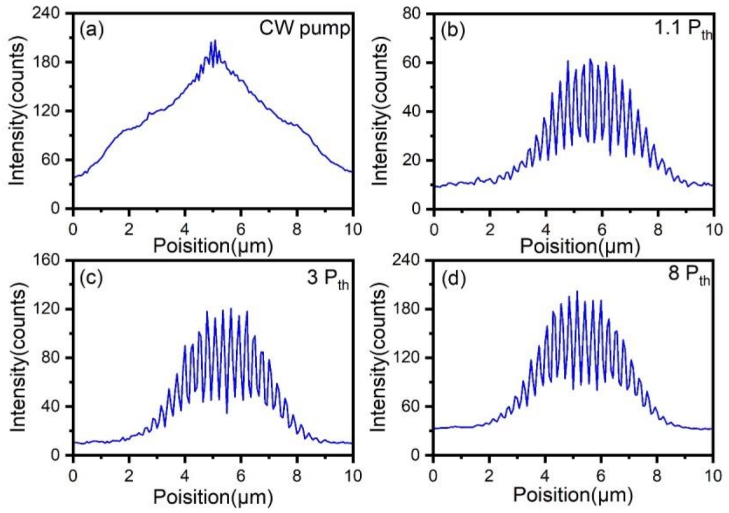

Figure S13. Fringe pattern of the data acquired by extracting from the interference pattern across the auto-correlation point at different intensity. (a) CW pump; (b)1.1 P_{th}; (c) 3 P_{th}; (d)8 P_{th}.

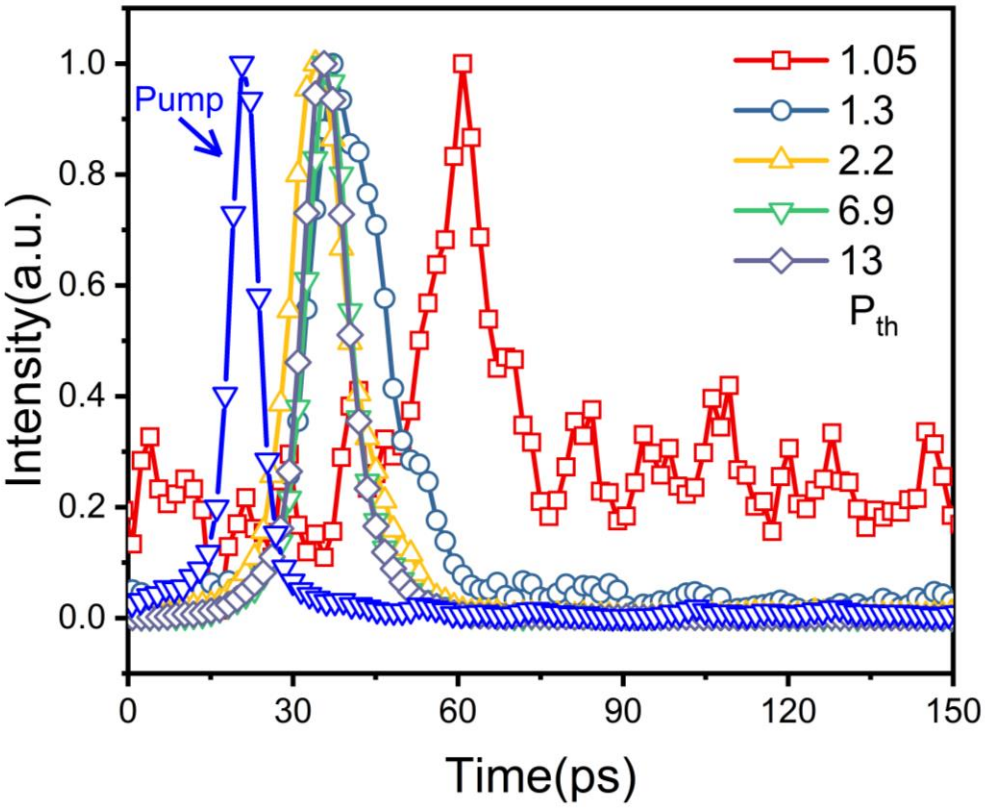

Figure S14. The decay curves of the ground-state emission extracted directly from the images of the streak camera.

References:

[1] S. Ithurria, G. Bousquet, B. Dubertret, *J Am Chem Soc.* **2011**, *133*, 3070.

[2] S. Ithurria, M. D. Tessier, B. Mahler, R. P. S. M. Lobo, B. Dubertret, A. Efros, *Nature Materials.* **2011**, *10*, 936.

[3] A. A. Rossinelli, A. Riedinger, P. Marques-Gallego, P. N. Knusel, F. V. Antolinez, D. J. Norris, *Chem Commun.* **2017**, *53*, 9938.

[4] H. Deng, H. Haug, Y. Yamamoto, *Reviews of Modern Physics.* **2010**, *82*, 1489.
